# Modeling and Design of SHPB to Characterize Brittle Materials under Compression for High Strain Rates

**DOI:** 10.3390/ma13092191

**Published:** 2020-05-10

**Authors:** Tomasz Jankowiak, Alexis Rusinek, George Z. Voyiadjis

**Affiliations:** 1 Institute of Structural Analysis, Poznan University of Technology, Piotrowo 5, 60-965 Poznań, Poland; tomasz.jankowiak@put.poznan.pl; 2 Laboratory of Microstructure Studies and Mechanics of Materials, UMR-CNRS 7239, Lorraine University, 7 rue Félix Savart, BP 15082, 57073 Metz CEDEX 03, France; rusinek1@univ-lorraine.fr; 3 Department of Mechanical Engineering, Chair of Excellence Universidad Carlos III de Madrid, Avda. de la Universidad 30, 28911 Leganés, Madrid, Spain; 4 Computational Solid Mechanics Laboratory, Louisiana State University, Baton Rouge, LA 70803, USA

**Keywords:** concrete, dynamic compression, Split Hopkinson Pressure Bars (SPHB), brittle materials, simulation

## Abstract

This paper presents an analytical prediction coupled with numerical simulations of a split Hopkinson pressure bar (SHPB) that could be used during further experiments to measure the dynamic compression strength of concrete. The current study combines experimental, modeling and numerical results, permitting an inverse method by which to validate measurements. An analytical prediction is conducted to determine the waves propagation present in SHPB using a one-dimensional theory and assuming a strain rate dependence of the material strength. This method can be used by designers of new SPHB experimental setups to predict compressive strength or strain rates reached during tests, or to check the consistencies of predicted results. Numerical simulation results obtained using LS-DYNA finite element software are also presented in this paper, and are used to compare the predictions with the analytical results. This work focuses on an SPHB setup that can accurately identify the strain rate sensitivities of concrete or brittle materials.

## 1. Introduction

Protection of buildings and structures in emergency situations is a key issue that needs to be addressed [1]. Prevention in such situations is the most important element of a protection system, especially in cases of critical infrastructure facilities. However, despite precautions, accidents and disasters sometimes occur. These are often of an urgent nature and can result in constructions (or their parts) being subjected to fast dynamic loads that correspond to high energy transfers with short loading times, which can lead to the damage or failure of a structure. Such situations may be related to the occurrence of a sudden load (e.g., a shock wave) caused by the blast of an explosive material or the impact of a bullet or some other object flying at a high velocity [2]. Predicting the effects of such a sudden load is particularly difficult because there is 100% certainty of the scale of damage and failure (and any possible preservation of building integrity) only after it has already occurred. Of course, it is possible in some instances to conduct experimental research on a smaller scale, but this only applies to selected structural elements [3]. Another solution is the use of advanced computer simulations that can accurately determine the dynamic behavior of a building and its structure in the event of a sudden dynamic load. However, the precision of calculations in these cases depend on many factors, such as the methods used in the calculations, how precisely the geometry of the structure under consideration was modeled and the interaction of components and behaviors of the materials from which the analyzed object was built. In the case of building structures, brittle materials such as glass or concrete are also often used for construction. The latter is often used particularly for the construction of reinforced concrete structural elements. The behavior of concrete, especially in the case of dynamic loads when high deformation speeds occur, is very complex [4]. This aspect—namely, how to determine the sensitivity of concrete during dynamic compression and then correctly determine the parameters of a constitutive model that could be used in concrete simulations—will be considered in detail in this work.

A description of dynamic material behavior is generally difficult to assess in the case of brittle materials. Different experimental techniques must be coupled in order to cover such a wide range of strain rates, as shown in Table 1 and reported in [5,6]. For example, using metallic materials or polymers, the specimen must have a length of a few millimeters when using Kolsky bars [6,7,8,9,10,11]. However, in the case of concrete, the length must be equal to tens of millimeters (~50 mm) in order to obtain a representative macroscopic behavior, due to the microstructure and inclusions size. In this work, the aggregate size is between 0.0075 to 5 mm and corresponds to specific cases of clay, sand and gravel with a maximum diameter of 5 mm. For this reason, in brittle materials such as concrete, strain rates are relatively reduced due to the length of the specimen. Moreover, as the material behavior [12,13,14] in compression is different in comparison with tensile one [12,15,16], different experimental setups are necessary. In this work, a technique based on dynamic compression is described that allowed the material behavior of brittle materials to be defined for a large range of strain rates varying from 1 to 1000 (s^−1^). The setup used was based on the Kolsky bar setup [7,8]. The equilibrium of the force impulses on both sides of the specimen during a test is crucial to use the elastic waves theory to determine the macroscopic behavior of the material sandwiched between the two elastic bars.

**Figure 1:**
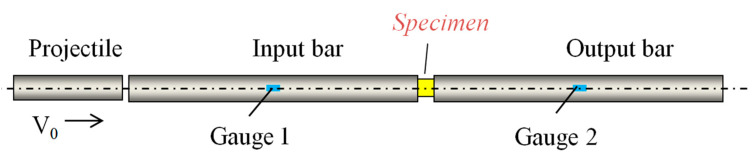
Schematic description of the split Hopkinson pressure bars (SHPBs) for dynamic compression.

A schematic description of a setup used for the brittle materials such as ceramic [17], glass or concrete is presented in Figure 1. Using this setup, it is possible to define dynamic material behaviors and strengths at high strain rates under compression [7,8]. This work presents an optimal configuration of the device by which to obtain the dynamic compressive strength of concrete at high strain rates close to 1000 (s^−1^), and this comprised a projectile and a concrete specimen sandwiched between two long elastic bars termed the input and output bars (see Figure 1). During the test, the projectile (striker) impacts the input bar with an initial impact velocity $V_{0}$, inducing an incident elastic wave. The compressive incident wave ($\sigma_{I},\varepsilon_{I}$) then propagates along the input bar with a velocity $C_{0}$. When the incident wave reaches the geometrical discontinuity between the input bar and the specimen, one part is reflected ($\sigma_{R},\varepsilon_{R}$) and one part is transmitted ($\sigma_{T},\varepsilon_{T}$), due to the difference of the mechanical impedance Z. Using the three wave measurements coupled to the theory of elastic waves [6,7,10,16], the average material behavior $\sigma \left(\varepsilon ,\dot{\varepsilon },T\right)$ and the dynamic compressive strength $f_{cd}\left({\dot{\varepsilon }}_{d}\right)$ for brittle materials can be obtained (this will be described in the next section). It should be noted that the waves were measured in the middle of the bars using resistance gauges to avoid the problem of wave superposition.

**Figure 2:**
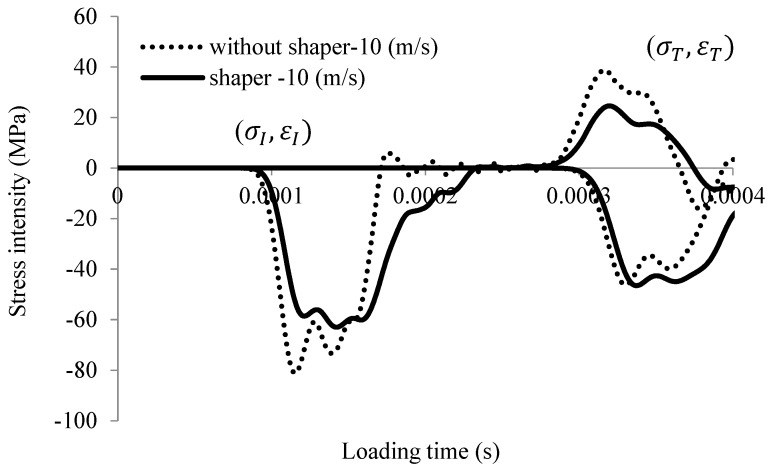
Elastic wave history signal using SHPB (numerical results with and without shaper) for an initial impact velocity of $V_{0}=10\;m/s$.

A typical three-wave measurement is reported in Figure 2. The pulse shaping method is not used in order to avoid smoothing the elastic wave signal and obtain a representative measurement related to the split Hopkinson pressure bars (SHPBs) [18,19,20,21]. It should be noted that a pulse shaper is frequently used to test brittle materials [20,21], as it increases the rising time and causes the strain rate to be both more constant and lower (see Figure 2). A comparison of waves for two examples (with and without a copper shaper) is presented in Figure 2. The material parameters for the copper are taken from [22]. The dimensions of the cylindrical copper shaper are as follows: 20-mm diameter and 1-mm thickness.

According to the CEB (Comité Euro-international du Béton) [23,24], the strength of concrete is highly strain-rate sensitive and can be defined using the compressive dynamic increase factor (CDIF) [23,24] as follows:

(1) $$CDIF\left({\dot{\varepsilon }}_{d}\right)=\frac{f_{cd}\left({\dot{\varepsilon }}_{d}\right)}{f_{cs}}=\{\begin{array}{cc} {\left(\frac{{\dot{\varepsilon }}_{d}}{{\dot{\varepsilon }}_{cs}}\right)}^{1.026\alpha } & if\;{\dot{\varepsilon }}_{d}\leq 30{\;s}^{-1}\\ \gamma \;{\dot{\varepsilon }}_{d}{}^{1/3} & if\;{\dot{\varepsilon }}_{d}>30\;s^{-1} \end{array}.$$

**Figure 3:**
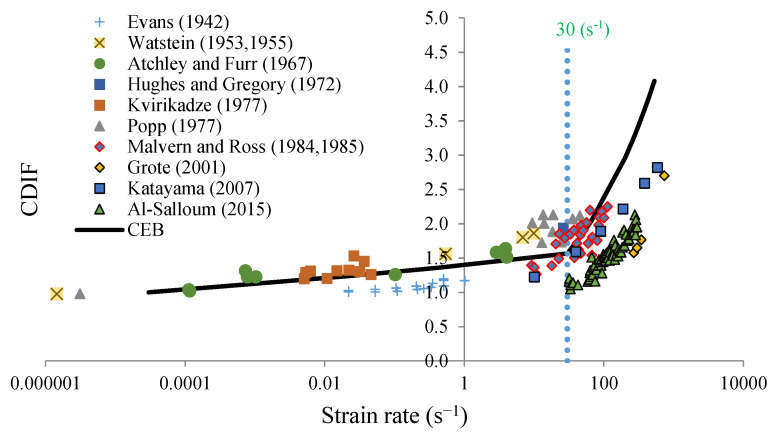
The compressive dynamic increase factor (CDIF) for concrete as a function of the strain rate.

In Equation (1), $f_{cd}$ is the dynamic compressive strength of concrete for a strain rate ${\dot{\varepsilon }}_{d}$ imposed on the material. The value ${\dot{\varepsilon }}_{cs}=0.00003s^{-1}$ is defined as a reference strain rate [23,24], and corresponds to the value used to obtain the quasi-static compressive strength of concrete $f_{cs}$. The parameters γ and α are defined by the CEB with $\gamma =10^{6.156\alpha -0.49}$ and $\alpha =1/\left(5+9f_{cs}/10\right)$. The values proposed by the CEB correspond to the experimental data reported by Bischoff and Perry (1991) [25]. The compressive dynamic increase factor (CDIF) is a function of strain rate (according to the CEB), as reported in Figure 3.

Additionally, experimental data have also been reported to define the strain rate sensitivity of concrete under compression [25], and data expressing different trends other than those identified by the CEB can be seen in Figure 3 [24,26,27]. Despite these results, the authors assume in this paper that concrete under dynamic compression behaves in accordance with CEB recommendations, which does not limit the whole analysis, because this trend is only one example. This is particularly important when using the presented analysis to study the dynamic strength of modern high-strength concrete or other brittle-like materials. The effect of strain rate sensitivity on the dynamic strength of concrete is quite high during compression when compared with its effect in other materials [6,11,21,22]. As a result, it is important to consider it to properly estimate the dynamic behavior of structures subjected to extreme impulsive loading [23].

## 2. SHPB Technique for Concrete—Analytical Description

To understand the real experimental measurements for the dynamic compression of concrete (a highly strain-rate-sensitive material), a new analytical technique should be used in comparison with compression of the metals [22]. The new analytical description should take into account the strain rate sensitivity of the material during the splitting of the incident wave into reflected and transmitted waves. The procedure used to estimate mechanical properties and errors during experiments may be estimated by coupling predictions based on Equation (2) with a full 3D numerical model that considers a nonlinear process not considered in the simple elastic wave theory. Assuming no dispersions of the elastic waves due to the Pochhammer–Chree effect related to the bar geometry (by considering an instantaneous rising time, which is not the case during experiments), the average mechanical behavior (stress–strain curve) and the compressive strength are defined using Equations (2) coupled o the three waves described previously in Figure 2:

(2-a) $$\dot{\varepsilon }\left(t\right)=\frac{2C_{A}}{L_{C}}\varepsilon_{R}\left(t\right)$$

(2-b) $$\varepsilon \left(t\right)=\frac{2C_{A}}{L_{C}}\int_{0}^{t}\varepsilon_{R}\left(t\right)dt$$

(2-c) $$\sigma \left(t\right)=E_{A}{\left(\frac{r_{A}}{r_{B}}\right)}^{2}\varepsilon_{T}\left(t\right)$$

All the necessary quantities including material parameters and geometry dimensions are defined explicitly in Table 2 and Table 3. Analyzing the previous equations, the average stress in the specimen σ(t) (Equation (2-c)) may be defined using the transmitted wave $\varepsilon_{T}\left(t\right)$. In addition, the reflected wave $\varepsilon_{R}\left(t\right)$ is used to calculate the strain rates $\dot{\varepsilon }\left(t\right)$ (Equation (2-a)) and strain ε(t) (Equation (2-b)) induced to the concrete specimen with time. During the experiment, it was clear that the strain rate was non-constant and depended on the hardening and strength of the material. As the deformation of concrete or other brittle materials is smaller than in metals or polymers, a short projectile $L_{P}$ can be used. It should be noted that the projectile length is proportional to the loading time of the specimen $t_{loading}=2L_{P}/C_{A}$. To make clearer all quantities defined for the calculations, the following indexes are used: aluminum (A), concrete (C) and steel (S), as reported in Table 2. Moreover, for concrete testing, the material used to design the bars and the projectile is frequently made of an aluminum alloy due its low mechanical impedance *Z* ($Z_{A}$ = 38.9 kg/s) (Equation (3-a)):

(3-a) $$Z_{A}=AC_{A}\rho_{A}$$

(3-b) $$Z_{C}=AC_{C}\rho_{C}$$

The value obtained for aluminum is close to the one for the concrete specimen ($Z_{C}$ = 22.7 kg/s) (Equation (3-b)). The value is obtained assuming a circular cross section $A={\pi r}^{2}$ with a radius of r = 30 mm. For comparison, the mechanical impedance of a steel bar is equal to $Z_{S}$ = 114 kg/s. Thus, the impedance of concrete is 1.71 times smaller than the impedance of an aluminum alloy and 5.04 times smaller than steel. Additionally, a subscript C means concrete, A refers to aluminum and S is steel. Therefore, the amplitude of the transmitted wave is reduced if steel bars are used to test the concrete material [19].

The incident stress $\sigma_{I}$ and the elastic strain intensity $\varepsilon_{I}$ can be determined if the initial impact velocity and the physical parameters of the bars are known [11,22]:

(4) $$\sigma_{I}=\frac{\rho_{A}C_{A}V_{0}}{2}\;\mathrm{and}\;\varepsilon_{I}=\frac{\sigma_{I}}{E_{A}}$$

The strain and stress values depend on the material properties of the input bar and of the projectile and its initial impact velocity $V_{0}$. The intensity of the transmitted wave $\varepsilon_{T}$ is calculated using Equations (1) and (5):

(5) $$\sigma_{T}=f_{cd}\left({\dot{\varepsilon }}_{d}\right){\left(\frac{r_{c}}{r_{A}}\right)}^{2}\;\;\mathrm{and}\;\varepsilon_{T}=\frac{\sigma_{T}}{E_{A}}$$

Finally, the reflected wave $\varepsilon_{R}$ is calculated as the difference between the incident (Equation (4)) and transmitted (Equation (5)) waves, assuming force equilibrium:

(6) $$\sigma_{R}=\sigma_{I}-\sigma_{T}\;\mathrm{and}\;\varepsilon_{R}=\frac{\sigma_{R}}{E_{A}}$$

The average strain rate and strain induced to the concrete specimen can be calculated as follows:

(7) $${\dot{\varepsilon }}_{d}=\frac{2C_{A}}{L_{C}}\varepsilon_{R}\;\mathrm{and}\;\varepsilon_{d}={\dot{\varepsilon }}_{d}\Delta t$$

The set of Equations (1)–(7) were solved considering the geometry of the bars, the projectile, the specimen and the initial impact velocity of the projectile. The values $f_{cd}$, ${\dot{\varepsilon }}_{d}$, $\varepsilon_{d}$, $\left(\sigma_{I},\varepsilon_{I}\right)$, $\left(\sigma_{R},\varepsilon_{R}\right)$ and $\left(\sigma_{T},\varepsilon_{T}\right)$ were calculated using the Newton–Raphson iterative algorithm.

**Figure 4:**
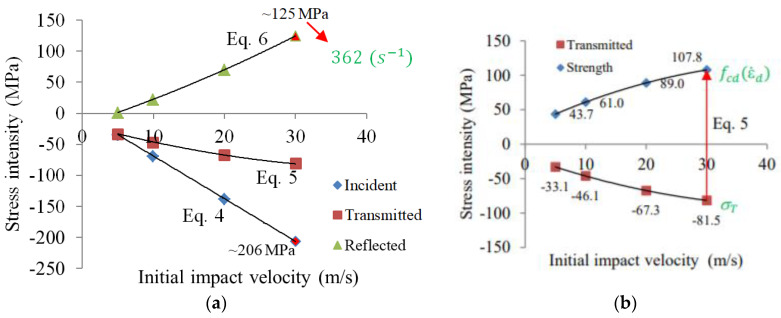
(**a**) Dependency of the incident, transmitted and reflected stress intensity for different initial impact velocities; (**b**) relation between the dynamic concrete strength and the intensity of the transmitted wave.

Based on the previous analytical description, the material parameters and the geometry of the setup and specimen (Table 2 and Table 3, respectively), it can be observed that the incident stress intensity increased with the initial impact velocity (Figure 4a). As reported before, at 30 m/s the stress level of the compressive wave was about 206 MPa (Figure 4a). This value must be smaller than the yield stress of the aluminum to avoid plastic deformation. The stress intensity in the specimen and its dynamic strength were calculated based on the transmitted wave amplitude using Equation (2-c). Equation (5) was used to predict the average value of the stress and strain if the transmitted wave in the middle of the output bar is known. Moreover, the reflected wave was used to calculate the strain rate and the strain in the specimen using Equation (2-a,b). To predict the average strain rate during the test, Equations (6) and (7) were used, and showed an increase in the strain rate tied to the initial impact velocity. As an example, for an initial impact velocity of $V_{0}$= 30 m/s, the stress intensity of the reflected wave was equal to 125 MPa, while the strain rate was close to 362 (s^−1^) (Equations (6) and (7), respectively).

Both trends shown in Figure 4a are nonlinear (i.e., transmitted wave and reflected wave; Equations (5) and (6), respectively). Only the intensity of the incident wave is linear (Equation (4)). It should be added that the limit of this simplified analysis is the yield stress of the input bar material. It is also important to note that real maximum stress intensities of the incident wave were higher during short periods of time than the theoretical one calculated for the Pochhammer–Chree effect [6,11,22].

The following section concerns designing the experimental setup, and it addresses what to change in order to obtain the maximum possible strain rate from the experiments. 

**Figure 5:**
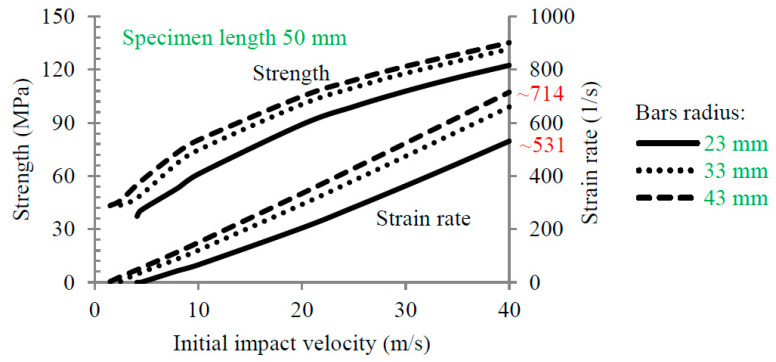
The effect of the initial impact velocity on the strength and strain rate for different radii of the transmitted bar (23, 33 and 43 mm).

**Figure 6:**
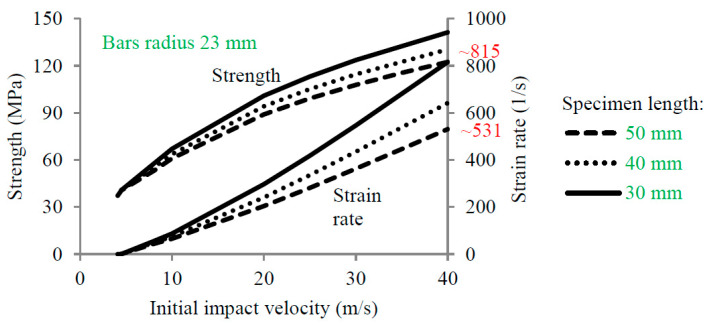
Effect of the initial impact velocity on the strength and strain rate for different lengths of the specimen (50, 40 and 30 mm).

It is well known that increasing the bar radius and assuming a constant specimen radius (20 mm in this work) increases the strain rate, as shown in Figure 5. For example, assuming a bar radius of 23 mm (1.15 times larger than the concrete specimen radius), the maximum strain rate is about 531 (s^−1^), while the maximum strain rate is about 714 (s^−1^) for a radius of 43 mm (2.15 times larger than the concrete specimen radius) (Figure 5). However, this technique of changing the bar diameter is not the most appropriate way to increase the strain rate. Based on the previous results, Figure 5, it is observed that the rising strain rate is faster with the diameter bar increase allowing to reach a bigger value for the strain rate for an imposed impact velocity ($\sim {714\;s^{-1}|}_{\emptyset =43\;\mathrm{mm}}^{40\;m/s}$ and ${\sim 531\;s^{-1}|}_{\emptyset =23\;\mathrm{mm}}^{40\;m/s}$). Moreover, it is possible to increase the strain rate by changing the initial length of the specimen. Based on the current configuration (SPHB with a bar diameter of 23 mm), the strain rate varies from 531 to 815 (s^−1^) if the specimen length decreases from 50 to 30 mm (Figure 6). By coupling the changing of both variables (using a bar radius of 43 mm and a short specimen of 30 mm), it is possible to reach a maximum strain rate close to 1166 (s^−1^). Concerning the analytical approach, the dynamic strength of concrete according to CEB was assumed to estimate the transmitted and reflected wave intensities. For a strain rate equal to 1166 (s^−1^), the CDIF is equal to 5.3 (strength 159 MPa). However, the size cannot decrease continuously for concrete, since the material behavior must be representative and the aggregate sizes must be considered. Thus, the specimen must be designed considering the representative elementary volume (REV) [28,29].

The above calculations and analysis should be used to design the setup geometry and its configuration in order to obtain the correct and expected stress levels or strain rates during experiments. It can be useful to test specific concrete types such as ultra-high strength concrete (UHSC) with a static compressive strength close to 100 MPa. The proposed analytical approximation can be used with success to predict the effects of a strong strain rate sensitivity on the material behaviors of concrete or other brittle materials in SHPB testing.

## 3. Simulation of the SPHB Technique for Material Characterization of Concrete at High Strain Rates

**Figure 7:**
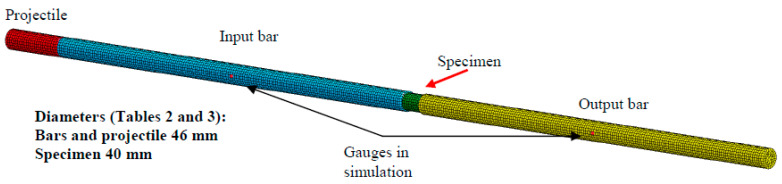
Discretization of the finite element model to simulate dynamic compression of concrete.

The following numerical simulations present how it is possible to describe the dynamic compressive strength and strain rate of concrete during dynamic failure using the SHPB technique. Previously neglected effects (dispersion of the wave and loading time) were revealed in the results of a full three-dimensional model. The simulation corresponding to the process of elastic wave propagation and related to the SHPB technique was performed using an explicit integration scheme in LS-DYNA [30]. All parts of the SPHB setup were considered: projectile, input and output bars, as well as the specimen. Their geometry and dimensions are presented in Figure 1 and reported in Table 3. The model was discretized by hexagonal eight-node finite elements constant stress, with a total number of 50,000 elements (Figure 7). The finite element length in the projectile, bars and specimen was 0.006 m, while the fine mesh size was 0.003 m. The simulations using the above model considered all additional effects [22,31], including the punching effect and geometrical dispersion of the waves. The elastic properties are reported in Table 2. In addition, in order to define the behavior of the specimen, a continuous-damage surface cap model was used [32,33,34]. This advanced model for concrete considers strain rate sensitivity. Contact with a friction coefficient equal to zero was assumed between all parts of the SHPB. The results of the simulation were intended for comparison with the simplified theory (Section 1), which is why a no-friction condition was assumed. In general, the authors acknowledge that friction conditions are very important in many dynamic tests (e.g., with SPHB for metals [22]). As an example, using the configuration described in this paper and considering friction values equal to μ=0.6 and μ=0, a stress increase of approximately 7% and a strain rate decrease of approximately 5% could be observed.

In addition, the continuous-damage surface cap model was used to simulate the concrete behavior and its properties at high strain rates. The parameters were obtained by calibrating a typical concrete; namely, C30 grade. A detailed description of the model is reported in [32,33,34]. In this paper, the assumptions of the model are presented and reported in Appendix A only considering the material parameters discussed in Section 3.

**Figure 8:**
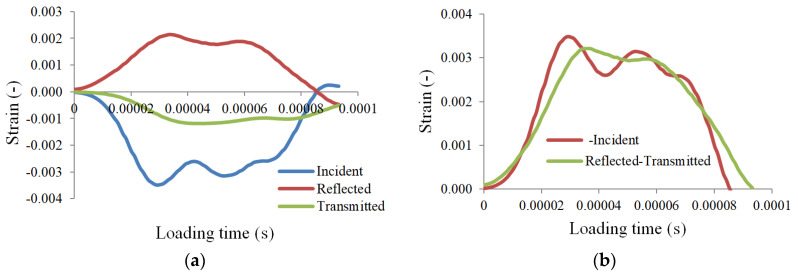
(**a**) Elastic waves measured during the dynamic compression of concrete with an impact v elocity 30 m/s (length of the specimen 0.05 m); (**b**) force equilibrium, input and output forces.

Based on the previous numerical solutions, experiments were mimicked and the same values were measured as the three elastic waves propagating along the two elastic bars. Before using the previous analysis (Equation (2-a–c)), the key point was to demonstrate that force equilibrium was reached during dynamic loading. In the following curves, 30 m/s was reported for impact velocity and 0.05 m was reported for length of the specimen in regards to the three waves after the shifting time to zero, Figure 8a. Based on this, a comparison can be done between the input and output forces. It can be observed that there was good agreement corresponding to force equilibrium (Figure 8b). It should be noted that force equilibrium was also reached also for other cases, including a shorter specimen with a length of 0.025 m.

**Figure 9:**
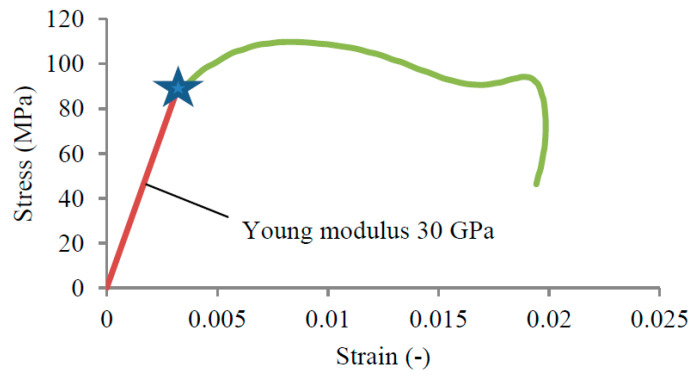
Material behavior description of concrete under dynamic compression based on the process of elastic wave propagation with an impact velocity of 30 m/s (strain rate 435 (s^−1^)).

Once the force equilibrium has been reached and achieved a conservation of energy and quantity of movement, the theory of elastic waves may be used to described the material behavior of concrete under dynamic loading. Even if the transmitted wave seems large, the strain applied stays relatively small, as shown in Figure 9. The strain was less than 2%, which demonstrates the brittle behavior of concrete. 

The material behavior (Figure 9), which was defined by applying the elastic waves theory to the elastic waves propagating along the bars (Figure 8), was in agreement with previous experiments performed on concrete (e.g., [24,35,36]).

## 4. Parametric Study Concerning the Main Crucial Material Parameters

Concerning all assumptions described in Appendix A, the most important to consider in this analysis is the strain rate sensitivity of concrete under compression. Additionally, the damage mechanism and regularization process by the fracture energy will be discussed and presented in the subsequent sections.

### 4.1. Analysis of Strain Rate Sensitivity in Compression

**Figure 10:**
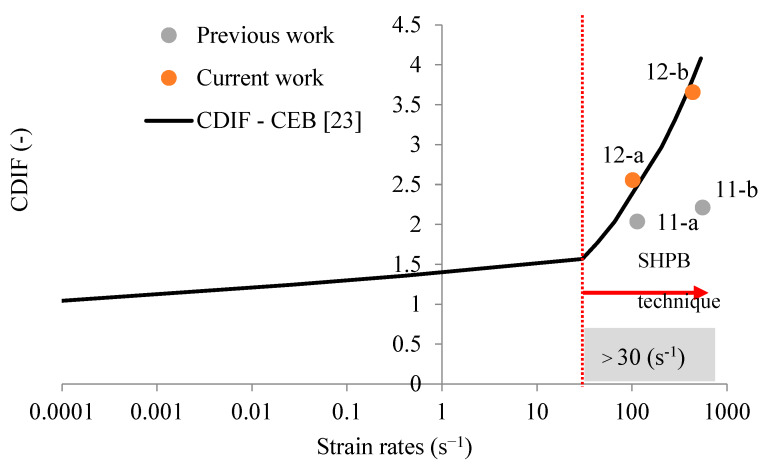
Comparison of the CEB recommendations with the strain rate sensitivity predicted for concrete by the continuous damage surface cap model.

The main parameters allowing the strain rate sensitivity of concrete under dynamic compression to be described are $\eta_{0c}$ and $N_{c}$ (see Appendix A). The usual values assumed for C30 concrete are $1.003\times 10^{-4}\;1/s$ and 0.78, respectively [32,33]. Using these values and simulating material behaviors under dynamic compression, it can be observed that the predicted dynamic strength of concrete is below the CEB recommendation (Figure 10) [23,25]. Thus, the dynamic behavior of concrete has been underestimated in compression at high strain rates [25]. The numerical model has shown a trend during experiments [24,26,27] that is different from the recommendation of the CEB (see Figure 3). The main parameters used to model the behavior of C30 concrete under dynamic compression are presented in Table 4.

**Figure 11:**
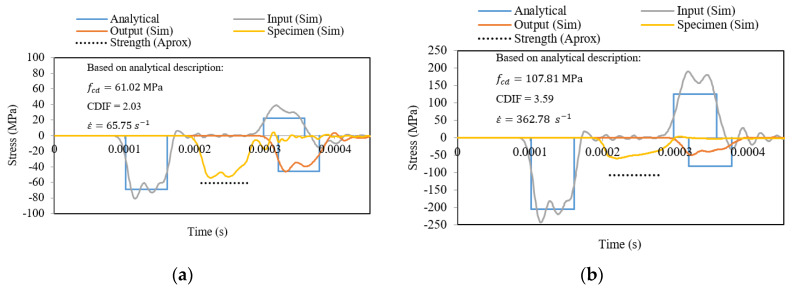
Waves in simulations of dynamic compression using SHPB for parameters from the previous work [32,33]; (**a**) 10 m/s, (**b**) 30 m/s.

**Figure 12:**
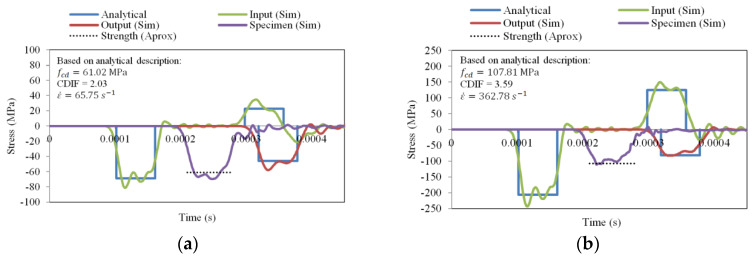
The waves in simulation of dynamic compression using SHPB for parameters from the current work; (**a**) 10 m/s, (**b**) 30 m/s.

The real strain rate sensitivity presented in Figure 10 must be used to correctly simulate the material behavior in order to later define the response of a structure designed with concrete. A more appropriate approximation by which to estimate the real behavior of concrete under dynamic compression for a large range of strain rates using the SHPB technique can be obtained for $\eta_{0c}=1.2\times 10^{-4}\;1/s$ and $N_{c}=0.58$ (Figure 10). The CEB approximation function is identified based on experimental results reported in Figure 10. In addition, the strain rates for which the SHPB technique is valid are shown. Points 11-a, 11-b, 12-a and 12-b in Figure 10 are obtained based on the numerical results defined in Figure 11a,b and Figure 12a,b. The strain rate and the stress level correspond to the maximum value of the signal reached on time.

The numerical simulations of the dynamic compression of concrete were analyzed in detail for two initial impact velocities: 10 and 30 m/s. The results of the numerical simulations were compared with the CEB [23], which was the best fit for several experimental results.

To avoid plastic deformation of the bars and to have pure elastic wave propagation, the yield stress of a bar was assumed equal to 250 MPa (see Figure 4a). Simulations were performed for both sets of constitutive parameters presented in Table 4. The results of the previous parameters for both impact velocities are presented in Figure 11, and for the results of the current set of parameters are shown in Figure 12. The wave intensity from the simulations in term of stress in the middle of the input and output bars (lines “Input (Sim)” and “Output (Sim)”, respectively) are compared to the analytical predictions described previously assuming an ideal rectangular elastic wave pulse (called “Analytical”). For the plots in Figure 10 and Figure 11, the stress history in the middle of the specimen (line “Specimen (Sim)”) is also shown based on the simulations. The dashed lines (which describe the dynamic strength according to the CEB, based on the previous analysis) are also presented (line “Strength (Approx)”). At a lower initial impact velocity of 10 m/s, the strength of concrete agreed for both sets of parameters (Table 4 and Figure 11a,b). For higher initial impact velocities, only the new set of parameters described in this current work allowed results to be obtained that were in agreement with the CEB recommendations (Figure 12b).

The analysis showed that material model parameter identification should be conducted using the geometry and complexity of the whole SPHB experimental setup (including the projectile, bars and specimen) in cases of brittle materials with a high strain rate sensitivity.

### 4.2. Analysis of Mesh Size Sensitivity

The pathological influence of finite element discretization (mesh size) is an important problem during concrete simulation due to the softening effect (decreasing of the stress with strain) [37,38,39]. This problem has been investigated, and several results and studies have been reported in several articles and books. The two methods of regularization for this phenomenon have been well acknowledged (on the level of mathematics or numerical formulation). The first group of regularization methods has included strain rate-dependent models in which the stress state depends on the speed of deformation [40,41]. This method is mainly used under dynamic loadings. This group also includes the Cosserat and micropolar models, which are used for soil and granular media descriptions [42]. To model damage and failure in concrete, non-local models may be used [43,44], as well as higher-order gradient models [45,46,47,48,49,50]. Non-local models have introduced an averaging function that converts local variables into non-local ones according to a specific weight function. There was a possibility to regularize the problem at the level of numerical formulation by explicitly introducing the width of plastic zone deformation localization in the finite element [51]. In addition, automatic re-meshing of finite elements has been used based on a local error caused by large gradients of internal variables. Discontinuity of the displacement field inside the finite element has also been introduced [52], and this has led to regularizing the problem. All the aforementioned methods of regularization have been introduced into the material model with an internal characteristic length scale.

**Figure 13:**
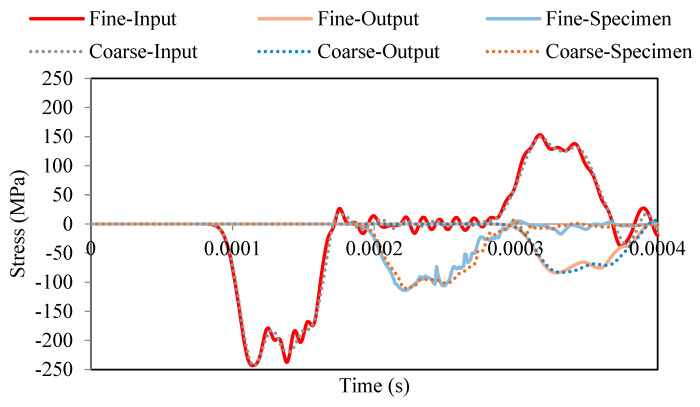
Mesh size sensitivity in a simulation of dynamic compression using SHPB for an initial impact velocity equal to 30 m/s.

For the calculations presented in this article, the authors used a material model with a fracture damage energy coupled to visco-plasticity (see Equations (A1)–(A9) in Appendix A) to regularize the solutions [33], and the important material parameters $G_{fc}$ and B (the fracture energy in uniaxial compression and the compression shape softening parameter, respectively) were considered. The values of these parameters for C30 are 6.838 MPa·mm and 100, respectively [32,33]. The simulations were done for two different finite element meshes. The coarse one was previously presented in Figure 7, and a fine one corresponded to the one-half element size. Finally, the size of the mesh in concrete was 6 or 3 mm (fine mesh), while it was 6 mm in the bars and projectile. The numerical results in term of elastic waves along the input and output bars are presented in Figure 13.

The mesh size sensitivity of the numerical results is acceptable (small enough) to assume that the results were not mesh size dependent. Based on these curves the behavior (stress-strain and strain-rate curves) for both cases was also similar. The model combined with the analyzed material model behavior was correct, and a mesh size dependency was not observed.

### 4.3. Analysis of the Fracture Energy in Compression Sensitivity

The stress-strain curve may vary for different kinds of concrete. The slope of the curve in the unloading part is crucial. It is important to know whether the curve can be measured using the SPHB technique. The main parameter measured up to now has been strength. However, the addition of some components as aggregates or fiber reinforcements in the concrete can change other mechanical properties. A parametric study was conducted to prove that potential changes in the softening curve shape of a material can be defined and observed during dynamic testing. The analysis was conducted by considering the changes of the two material parameters $G_{fc}$ and B (Table 5). During the simulations the values from Table 5 were used: Example 1 concerned the default values of both parameters; in Example 2, the value of the fracture energy in uniaxial compression $G_{fc}$ was divided in two; in Example 3, the value was defined using a default fracture energy, but the compressive shape softening parameter B was 10 times smaller; and finally, in Example 4, both changes were used in the simulations.

**Figure 14:**
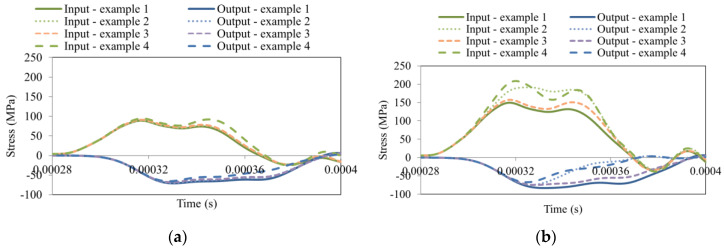
Parametric study concerning $G_{fc}$ and B on the process of dynamic compression using SHPB for an initial impact velocity equal to (**a**) 20 m/s and (**b**) 30 m/s.

The results corresponding to all previously described examples are reported in Figure 14a,b. For better visibility, only the transmitted and reflected waves are presented in the figure. It is clear that by decreasing the fracture energy $G_{fc}$ and the compressive shape softening parameter *B*, the material starts to become more brittle.

All effects are reported in Figure 14 in terms of elastic wave propagation. As can be observed, the material behavior definition based on the analysis of wave propagation changed depending on the parameters used (Table 5). The incident wave was not affected, since it is related to the initial velocity, the length of the projectile and the mechanical properties of the input bar. The analysis has shown that if the softening of the specimen changes (increasing: material becomes more brittle; decreasing: material becomes more ductile) then it is possible to see the effect on the elastic waves. The effect is visible on the transmitted and the reflected waves. The behavior in Example 4 is more brittle because the stress intensity is lower than in Example 1. This is observable especially for a higher impact velocity (30 m/s; Figure 14b) than for a lower impact velocity (20 m/s; Figure 14a). This is the main reason that the stress intensity of the reflected wave (strain rate of the test) was higher. 

## 5. Conclusions

The dynamic behavior of concrete during impact or a blast is very often analyzed using numerical simulations. During these kinds of loadings, high strain rates are reached and observed in the material. To predict the material’s behavior and dynamic strength, very precise tests and dynamic measurements are necessary, as has been discussed in this paper. Experimental results are then used to calibrate the concrete material model parameters. If the initial boundary value problem is used to simulate the dynamic behavior of the structure with the correct material model, its prediction will agree with the experimental observations. The following main concepts and results are presented in detail in this article:

- An analytical solution to predicting stress and strain wave intensities was presented. This could be used to simplify the design process of SHPB and check consistency in further experimental test results.
- The effect of the initial impact velocity of the projectile on the strength and strain rate reached in the specimen was determined for different bar diameters.
- A method to calibrate the material model for concrete including strain rate sensitivity was presented. A numerical simulation was used to find a correct value of the parameters that define the strain rate sensitivity. As discussed, the original parameters have very low values of dynamic strength for compressed concrete in comparison with the analytical solution.
- The presented analysis proved that the solution was not sensitive to mesh size. The important aspect is that possible changes in fracture energy during compression or the shape of the softening (descending) part of the curve can be identified using this experimental technique.
- This work assumes that the concrete specimen is in equilibrium during the simulations, and that the friction coefficient has limited influence on the final results.

For each case considered in this work, the previous dynamic experimental results recommended by the CEB as CDIF were compared with the numerical results. This work may be used to summarize the design process of SHPB for concrete to reach a certain strain rate. Extension of the analysis to other classes of concrete (concrete C30 was assumed) or glass is possible using the same procedure. These new materials (e.g., ultra-high performance concrete) can also be tested using the setup described herein. However, a limitation is imposed not to exceed the yield stress of the Hopkinson bars.

## Figures and Tables

**Table 1:** Experimental techniques for specific strain rates.

Experimental Technique	Strain Rates in Metals (s^−1^)	Strain Rates in Brittle Materials (s^−1^)
Servo-hydraulic machines	10^−6^ to 10^0^	
Specialized machines	10^0^ to 10^2^	
Conventional Kolsky bar	10^2^ to 10^4^	10^1^ to 10^3^
Miniaturized Kolsky bar	10^4^ to 10^5^	
Plate impact	10^5^ to 10^7^	

**Table 2:** Material parameters of the experimental setup from Figure 1.

Material	Value (Units)
Aluminum alloy	
Young’s modulus, $E_{A}$	70,000 (MPa)
Density, $\rho_{A}$	2700 (kg/m^3^)
Elastic wave speed, $C_{A}=\sqrt{E_{A}/\rho_{A}}$	5091.8 (m/s)
Concrete C30/37	
Young’s modulus, $E_{C}$	26,357 (MPa)
Density, $\rho_{C}$	2450 (kg/m^3^)
Elastic wave speed, $C_{C}=\sqrt{E_{C}/\rho_{C}}$	3280.0 (m/s)

**Table 3:** Geometry dimensions of the experimental setup from Figure 1.

Part	Value (Units)
Bars and projectile	
Input and output bar length, $L_{A}$	1 (m)
Length of the projectile, $L_{P}$	0.15 (m)
Radius of the projectile and bars, $r_{A}$	0.023 (m)
Specimen	
Length of the specimen, $L_{C}$	0.05 (m)
Radius of the specimen, $r_{C}$	0.02 (m)

**Table 4:** Main parameters used to predict the dynamic properties of concrete.

Parameter	Value (Units)
Articles [32,33]	
Fluidity in compression, $\eta_{0c}$	0.0001003 (s^−1^)
Power in compression, $N_{c}$	0.78 (-)
Current analysis	
Fluidity in compression, $\eta_{0c}$	0.00012 (s^−1^)
Power in compression, $N_{c}$	0.58 (-)

**Table 5:** Main parameters used to predict the fracture energy sensitivity.

Examples	Parameter	Value (Units)
Example 1 (default)	Fracture energy in uniaxial compression, $G_{fc}$	6.838 (MPa·mm)
	Compressive shape softening parameter, B	100 (-)
Example 2	Fracture energy in uniaxial compression, $G_{fc}$	3.419 (MPa·mm)
	Compressive shape softening parameter, B	100 (-)
Example 3	Fracture energy in uniaxial compression, $G_{fc}$	6.838 (MPa·mm)
	Compressive shape softening parameter, B	10 (-)
Example 4	Fracture energy in uniaxial compression, $G_{fc}$	3.419 (MPa·mm)
	Compressive shape softening parameter, B	10 (-)

## Appendix A

The following explanation of the constitutive model for concrete is presented mainly based on the literature [32,33,34]. The authors present only the most important assumptions of the model discussed and analyzed in Section 3 of this paper.

The first assumption is that shear and the bulk moduli allow one to describe the elastic state of the material considered. The plasticity model depends on the first invariant of the stress tensor $J_{1}$, as well as on the second $J_{2}^{\prime }$. For triaxial compression, the plastic potential is limited by the cap hardening function. The yield surface describes the shear failure limited to torsion and triaxial tension by the Rubin scaling function [32,33,34].

Using a visco-plastic formulation, it is possible to consider the strain rate sensitivity of a material. Thus, a visco-plastic stress tensor without damage $\sigma_{ij}^{vp}$ is defined as follows:

(A1) $$\sigma_{ij}^{vp}=\left(1-\gamma \right)\sigma_{ij}^{T}+\gamma \sigma_{ij}^{p}$$

where $\sigma_{ij}^{T}$ is the trial elastic stress and $\sigma_{ij}^{p}$ is the inviscid plastic stress tensor. The viscous variable γ is calculated using Equation (A2):

(A2) $$\gamma =\frac{\Delta t/\eta }{1+\Delta t/\eta }$$

where η is the effective fluidity coefficient, which is calculated independently in compression (or in shear) and in tension using the following formulation:

(A3) $$\begin{array}{c} \begin{array}{cc} \eta =\eta_{s}+{\left(\frac{J_{1}}{\sqrt{3J_{2}^{\prime }}}\right)}^{pwrc}\left(\eta_{c}-\eta_{s}\right) & \mathrm{compression} \end{array}\\ \begin{array}{cc} \eta =\eta_{s}+{\left(\frac{-J_{1}}{\sqrt{3J_{2}^{\prime }}}\right)}^{pwrt}\left(\eta_{t}-\eta_{s}\right) & \;\mathrm{tension} \end{array} \end{array}$$

where $\eta_{t}$, $\eta_{c}$ and $\eta_{s}$ are calculated as follows:

(A4) $$\eta_{t}=\frac{\eta_{0t}}{{\dot{\varepsilon }}^{N_{t}}},\eta_{c}=\frac{\eta_{0c}}{{\dot{\varepsilon }}^{N_{c}}}\;\mathrm{and}\;\eta_{s}=S_{rate}\eta_{t}$$

where the material parameters $\eta_{0t}$, $\eta_{0c}$, $N_{t}$, $N_{c}$, $S_{rate}$, pwrc and pwrt describe the strain rate sensitivity of the material model, and the variable $\dot{\varepsilon }$ describes the effective strain rate in the material.

Using the continuous damage surface cap model [32,33,34], the scalar damage variable d is used to describe the stress tensor with damage $\sigma_{ij}^{d}$ based on the visco-plastic stress tensor without damage $\sigma_{ij}^{vp}$ as follows:

(A5) $$\sigma_{ij}^{d}=\left(1-d\right)\sigma_{ij}^{vp}$$

Two independent damage mechanisms exist related to compression (or shear) and tension. Both are initiated by plasticity, since the initial damage surface coincides with the plastic shear function [33]. Therefore, two energetic criteria are used to describe the damage initiation (Equation (A6)) and its evolution (Equation (A7)).

The damages are initiated when the energy-type terms $\tau_{c}$ (in compression) and $\tau_{t}$ (in tension) exceed a damage threshold defined as $r_{0c}$ or $r_{0t}$. The first invariant of the stress tensor $J_{1}$ is related to the compressive or tensile state, as follows:

(A6) $$\begin{array}{cccc} \tau_{c}=\sqrt{\frac{1}{2}\sigma_{ij}\varepsilon_{ij}} & \mathrm{if} & \{\begin{array}{c} J_{1}\\ \tau_{c}\geq r_{0c} \end{array} & \begin{array}{c} \mathrm{compression}\\ \mathrm{energy}\; \end{array}\\ \tau_{t}=\sqrt{E\varepsilon_{max}^{2}} & \mathrm{if} & \{\begin{array}{c} J_{1}\\ \tau_{t}\geq r_{0t} \end{array} & \begin{array}{c} \mathrm{tension}\;\\ \mathrm{energy}\; \end{array} \end{array}$$

The damage initiations are dependent on the elastic-plastic stress tensor $\sigma_{ij}$ and strain tensor $\varepsilon_{ij}$, or to the maximum main strain $\varepsilon_{max}$ for the case corresponding to tension. The damage thresholds are calculated using the internal procedure of LS-DYNA.

Two softening functions d are used independently related to compression (or in shear) and tension:

(A7) $$\begin{array}{cc} d\left(\tau_{c}\right)=\frac{d_{max}}{B}\left[\frac{1+B}{1+Be^{-A\left(\tau_{c}-r_{0c}\right)}}-1\right] & \mathrm{compression}\\ d\left(\tau_{t}\right)=\frac{0.999}{D}\left[\frac{1+D}{1+De^{-C\left(\tau_{t}-r_{0t}\right)}}-1\right] & \mathrm{tension} \end{array}$$

where A and C are equal to the characteristic finite element length $l_{ch}$. Moreover, the variable A may be reduced as reported in Equation (A8). The internal parameter $d_{max}$ is the maximum damage level:

(A8) $$A=A{\left(d_{max}+0.001\right)}^{pmod}$$

where parameters B, D and pmod describe the shape of the softening functions in compression and in tension.

This model allows one to assure the uniqueness of the solution, which does not depend on mesh size and allows the problem to be regularized using material softening. The fracture damage energy $G_{f}$ depends on the damage mechanism (state of stress):

(A9) $$\begin{array}{c} \begin{array}{cc} G_{f}=G_{fs}+{\left(\frac{J_{1}}{\sqrt{3J_{2}^{\prime }}}\right)}^{pwrc}\left(G_{fc}-G_{fs}\right) & \mathrm{compression} \end{array}\\ \begin{array}{cc} G_{f}=G_{fs}+{\left(\frac{J_{1}}{\sqrt{3J_{2}^{\prime }}}\right)}^{pwrt}\left(G_{ft}-G_{fs}\right) & \mathrm{tension} \end{array} \end{array}$$

where $G_{fs}$, $G_{ft}$ and $G_{fc}$ describe the fracture energy in shear, tension and compression, respectively; the coefficients pwrc and pwrt are the parameters used to describe the transition from shear to tension and from shear to compression [33], and have the same values as in Equation (A3). The fracture damage energy also depends also on the strain rate. All material parameters for concrete C30 have been previously reported (e.g., [32,33,34]). In Section 3, only the parameters strongly influencing the results are discussed.
